# Creating animated medical images (Part 1)

**DOI:** 10.2349/biij.2.2.e32

**Published:** 2006-04-01

**Authors:** NA Kadri, MG Raha

**Affiliations:** Department of Biomedical Engineering, Faculty of Engineering, University of Malaya, Kuala Lumpur, Malaysia

## INTRODUCTION

With the advent of the Internet and World Wide Web (WWW), different image format became available. Some were static (GIF, JPEG) while others are animated (animated GIF, MPEG, Shockwave). Each carries distinct advantages for a given need and purpose, particularly for efficient transmission over the network. For instance, a GIF file is highly suitable for encoding diagram-like images – images that consist of line art and flat colour, whereas a JPEG is the chosen format for encoding images with photographic quality [1].

In encoding animated images, an animated GIF would be the chosen format for encoding a simple animation generated by a sequence of vector graphics while an MPEG format is preferred if animation with a movie-like quality is desired. Both these formats employ different algorithms to achieve the best animation quality at the optimal file size for network transmission [2].

Although the initial objective of developing the network, which later formed the framework for today’s Internet, was military in nature, the interconnectivity of these vast computing resources promises to be of much use for general users as well [3]. One of the driving factors behind the development of various applications and protocols, including the WWW, is the possibility of unlimited sharing of resources. The progress of the WWW has spawned a myriad of applications in numerous areas, be it for educational, research, or recreational purposes [4]. For instance, in the area of medical education the use of animation as a teaching tool has been proven to be one of the most effective methods in delivering learning materials [5,6]. Just by having a web browser, which incidentally can be acquired free of charge, the advantages of learning via animation may be shared with a large number of users.

This tutorial will try to document a few simple steps involved in producing animated images from a series of medical images, using widely available software. The image samples (in both GIF and JPEG formats) from a computed tomography (CT) study are also available for download.

## SOFTWARE

This two-part tutorial covers the use of applications that create animated GIFs, with the exception of MS PowerPoint, which produces animation that may only be used in a .ppt or .pps file. Animated GIF is chosen because of its cross-browser viewing capability and small file size (depending on the original source images).

Part 1 of this tutorial will focus on the use of proprietary software, while Part 2 will discuss the use of shareware and freeware applications that can be downloaded from the Internet. The list of software used in Part 1 is as follows:

- Proprietary – MS PowerPoint (Microsoft Corp., Redmond, WA, USA)
- Proprietary – Adobe ImageReady (Adobe Systems Inc., San Jose, CA, USA)

The discussion is by no means exhaustive and readers are encouraged to look up the respective vendor websites, or the various online tutorial websites dedicated to these software.

To fully utilize the examples in this tutorial, a series of sequential images are needed. One may source these images themselves or download directly from this hyperlink: http://www.biij.org/2006/2/e32/images.zip (923 KB). The ZIP file contains two folders containing 15 sequential JPEG and GIF images respectively, taken from a CT study at the University of Malaya Medical Centre, Malaysia (courtesy Prof. KH Ng).

The ZIP file has to be ‘unzipped’ before the images may be used. A popular utility is WinZip (WinZip International LLC, Mansfield, CT, USA), and an evaluation version may be downloaded at no charge from the website (http://www.winzip.com) or Download.com (http://www.download.com). The image files will be located inside two folders named “sample jpg” and “sample gif”, and this tutorial will assume that the ZIP file is unzipped at the **c:\** directory. All the examples used in this tutorial have been tested using Windows® XP Professional Edition® (Microsoft Corp., Redmond, WA, USA) and may still work on other versions of Windows® operating systems.

## MS POWERPOINT

MS PowerPoint is the most commonly used presentation software on the PC platform. Depending on the release version, it has a number of effects that can be used to create animation.

Step 1 – Open MS PowerPoint, and click on **Insert > Pictures > From File…**

**Figure 1:**
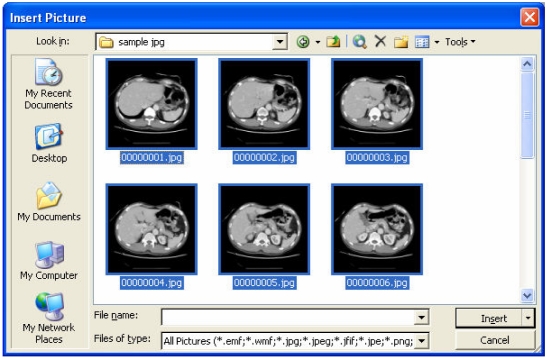
Selecting JPEG images to be inserted.

Step 2 – Select the **c:\sample jpg\** folder, and select all images within the folder by clicking **Ctrl+A** (Figure 1). Click **Insert** when finished.

**Figure 2:**
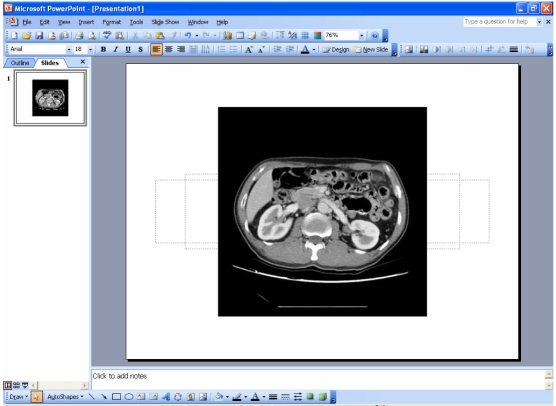
Images aligned at the centre of the slide.

Step 3 – The selected images are inserted into the slide; arranged diagonally from left to right (Fig. 2). The images now need to be aligned at the centre of the slide. To do this, select all images (**Edit > Select All** (or **Ctrl+A**)) and click **Draw > Align or Distribute > Align Center**, followed by **Draw > Align or Distribute > Align Middle** from the Drawing toolbar. Ensure that **Draw > Align or Distribute > Relative to Slide** is checked. The images should appear as one and aligned at the centre of the slide(Fig. 2).

**Figure 3:**
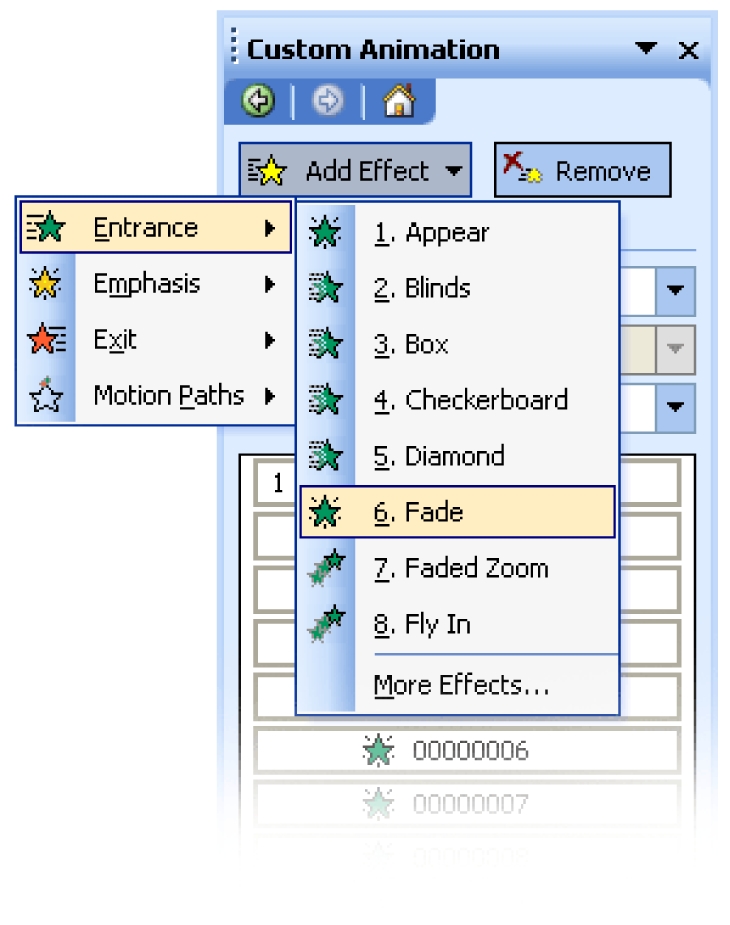
Selecting the animation effects.

Step 4 – To create the animation, click **Slide Show > Custom Animation…**, and the Custom Animation task pane will appear on the right side of the screen. The types of animation available depend on the MS PowerPoint version used, and this tutorial uses the “Fade” effect. To do this, select all images (**Edit > Select All** (or **Ctrl+A**), and click **Add Effect > Entrance > Fade**(Fig. 3). If “Fade” is not on the list, click on **More Effects…**

Step 5 – To produce a smooth transition between the images, customise the animation by modifying the “Fade” effect. At the Custom Animation task pane, select **After Previous** for the **Start** option; and select **Very Fast** for the **Speed** option.

Step 6 – The animation is now ready, and may be viewed by clicking **Slide Show > View Show** (or **Ctrl+F5**). An example of the MS PowerPoint file produced may be viewed at http://www.biij.org/2006/2/e32/sample.pps (546 KB).

Since MS PowerPoint is capable of handling multiple image formats, including GIF, JPEG, TIFF, PNG, any sequential images of these formats may be animated using the above steps. The final file size depends on the number of images used, and the original file size of the inserted images.

The above tutorial is written based on MS PowerPoint 2003 version, but may still be used with other versions of MS PowerPoint with very slight alterations.

## ADOBE IMAGEREADY

This proprietary software is from Adobe Systems, and availableas a package with Adobe Photoshop. It is primarily used to produce optimized images for web pages, including selective compression for JPEG, GIF, animated GIF, rollover images, and image maps. This tutorial will only focus on producing an animated GIF using the sample images provided.

Step 1 – Open Adobe ImageReady, and click on **File > Import > Folder as frames…**

**Figure 4:**
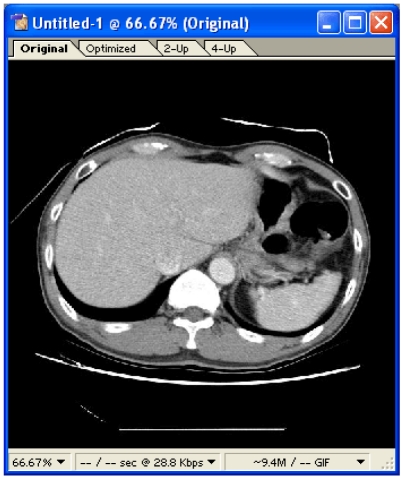
New document containing the series of images.

Step 2 – Select the **c:\sample jpg\** folder, and a new document is displayed (Fig. 4). This document actually contains the sequential images arranged in layers. A smaller pane located below the document displays the sequence of the images.

**Figure 5:**
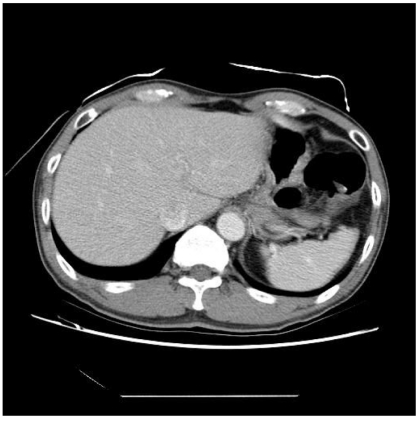
The animated GIF produced.

Step 3 – To produce an animated GIF, click **File > Save Optimized As…**, and select the destination. Click **Save** when finished. Figure 5 shows the resulting animated GIF.

One may preview the output in any web browser before producing the animated GIF by clicking **File > Preview In > Internet Explorer** (or **Ctrl+Alt+P**). Users may add additional web browsers by clicking **File > Preview In > Other…**

The dimension of the resulting animated GIF depends on the original image dimensions. To reduce the final file size, users may reduce the dimension of the document by resizing the document. Click **Image > Image Size…** and enter the new dimensions in pixel values.

The above tutorial is written based on Adobe ImageReady 7.0 version, but may still be used with other versions of Adobe ImageReady with very slight alterations.
